# Influence of Endosperm Starch Composition on Maize Response to *Fusarium temperatum* Scaufl. & Munaut

**DOI:** 10.3390/toxins14030200

**Published:** 2022-03-08

**Authors:** Marcin Wit, Piotr Ochodzki, Roman Warzecha, Emilia Jabłońska, Ewa Mirzwa-Mróz, Elżbieta Mielniczuk, Wojciech Wakuliński

**Affiliations:** 1 Department of Plant Protection, Warsaw University of Life Sciences, Nowoursynowska 159, 02-776 Warsaw, Poland; emilia_jablonska@sggw.edu.pl (E.J.); ewa_mirzwa_mroz@sggw.edu.pl (E.M.-M.); wojciech_wakulinski@sggw.edu.pl (W.W.); 2 Department of Applied Biology, Plant Breeding and Acclimatization Institute—National Research Institute, Radzików, 05-870 Błonie, Poland; p.ochodzki@ihar.edu.pl (P.O.); r.warzecha@ihar.edu.pl (R.W.); 3 Department of Plant Protection, University of Life Sciences in Lublin, Leszczyńskiego 7, 20-069 Lublin, Poland; elzbieta.mielniczuk@up.lublin.pl

**Keywords:** *Fusarium* ear rot, amylose, mycotoxin

## Abstract

*Fusarium temperatum* Scaufl. & Munaut is a newly described taxon belonging to the *Fusarium fujikuroi* species complex (FFSC) and a frequent causative factor of maize ear rot. The aim of the present study was to determine the responses to the disease in maize populations differing in endosperm features that were classified to flint, dent, and a group of plants with intermediate kernel characteristics. In inoculation studies, substantial variation of host response to the fungus was found among the tested maize types. The dent-type kernels contained significantly less amylose (28.27%) and exhibited significantly higher rates of infection (I_FER_ = 2.10) and contamination by beauvericin (7.40 mg kg^−1^) than plants of the flint maize subpopulation. The study documents a significant positive correlation between the *Fusarium* ear rot intensity (I_FER_) and ergosterol content (the R value ranged from 0.396 in 2015 to 0.735 in 2018) and between I_FER_ and the presence of beauvericin (the R value ranged from 0.364 in 2015 to 0.785 in 2017). The negative correlation between (I_FER_) and amylose content (ranging from R = −0.303 to R= −0.180) stresses the role of the endosperm starch composition in the kernel resistance to *Fusarium* ear rot. The conducted study indicated that the risk of kernel infection and contamination with fungal metabolites (beauvericin and ergosterol) was associated with the maize type kernels.

## 1. Introduction

*Fusarium* ear rot (FER) is considered one of the most important threats in the production of maize worldwide. The key causative agent of the disease is *Fusarium verticillioides* belonging to the *Liseola* section, according to the pre-molecular approach of Nelson *Fusarium* taxonomy system and, based on phylogenetic inference, included in the *Fusarium fujikuroi* species complex (FFSC). The etiology of *Fusarium* ear rot is much more complex [1]. In addition to *F. verticillioides*, many other FFSC taxons are reported as the causes of FER, e.g., *F. subglutinans* and *F. proliferatum.*

Although the occurrence of FFSC species is widespread [2,3], they are particularly important and destructive maize pathogens in areas with high temperatures and dry weather conditions during the maize flowering period [4]. The population of FER causative factors has been recently completed with new *Fusarium* taxons: *Fusarium kyushuense* [5], *Fusarium andiyazi* [6], *Fusarium boothii* [7], *Fusarium meridionale* [8], *Fusarium sacchari* [9], and *Fusarium temperatum*, which is phenotypically very close to *F. subglutinans* [10]. Since 2011, the occurrence of *F. temperatum* has been reported in Belgium, Spain, France, China, Indonesia, South Africa, Argentina, Costa Rica, and commonly in Poland [6,10,11,12,13,14,15,16,17]. The fungus belongs to the *Fusarium fujikuroi* species complex (FFSC) and represents the American clade of this complex [18,19,20,21,22,23]. Most recently, the genome of the species has been sequenced [24]. Similarly to other FFSC species, *F. temperatum* is toxigenic and has been recognized as a producer of beauvericins and enniatins. Both groups of secondary metabolites are cyclic hexadepsipeptides and, like other peptolides, are biosynthesized by non-ribosomal peptide synthetases (NRPS) with the participation of polyketide synthase (PKS) or fatty acid (FA) synthase [25]. The biological properties of these compounds are diverse and include antimicrobial, cytotoxic, and enzyme inhibition activity as well as oxidative stress induction [26].

The increasingly common occurrence of the species in the temperate climate zone of Europe and the emerging risks of cereal contamination with beauvericins [27] were the reasons to start the studies on maize infection with *F. temperatum* and contamination of kernels with hexadepsipeptides in relation to the endosperm starch composition in flint and dent maize. Flints and dents are the main types of maize currently cultivated worldwide, and their gene pools constitute major contributors in most of the maize breeding programs. The essential difference between these two types is the endosperm composition. This triploid tissue is the largest part of kernels, constituting an average of 85% of their weight. Its main component is starch composed mainly of two carbohydrate biopolymers: amylose (a water-soluble compound made up of long linear chains containing 1000 d-glucose units) and amylopectin (composed of d-glucose linear chains connected with three types of branch chains). The ratio of amylose and amylopectin significantly modifies starch resistance to enzymatic digestion and the presence of hard endosperm (grain hardness); both traits may influence host response to infection. Flint kernels are usually smaller in size than the dent type [28]. They have thick, hard, and vitreous endosperm in the outer (upper and side) layer of the kernel surface [29] characterized by lower maximum water content [30]. Although flint maize produces lower yields [28], it is a source of valuable genes for early vigor, cold tolerance [31], and adaptation to shorter vegetation periods [32]. Dent maize is characterized by the presence of a dent-shaped crown of kernels whose formation is the result of water loss by the soft starch endosperm during maize maturation. In breeding programs, an advantage of dent maize is their high productivity.

## 2. Results

### 2.1. Fusarium Ear Rot Infection

**Figure 1:**
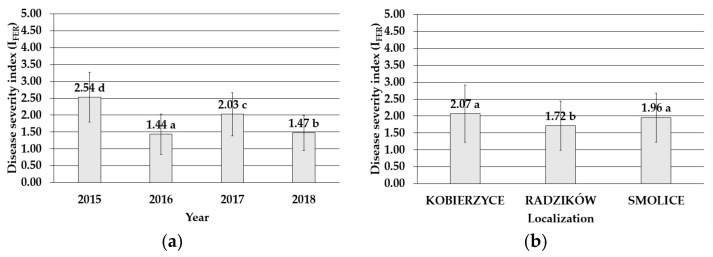
Mean value with standard deviation bars of the disease severity index of maize genotypes inoculated with *Fusarium temperatum* in the field test (**a**) during the four cropping seasons (2015–2018) and (**b**) during the four cropping seasons (2015–2018) in Smolice, Radzików, and Kobierzyce. The average values obtained within an individual cropping season for all genotypes of maize marked with the same letter do not differ significantly at *p* ≤ 0.05. The average values obtained for an individual localization over the all years of study marked with the same letter do not differ significantly at *p* ≤ 0.05.

The four-year study performed in three locations (Radzikow, Kobierzyce, and Smolice) in 2015–2018 emphasized the role of *Fusarium temperatum* as a causative factor of *Fusarium* ear rot. The ears of maize exhibited various degrees of infection from 0 in some circumstances to 5 points according to a 6-point disease severity scale. The mean value of the disease severity index (I_FER_) varied from I_FER_ = 1.44 in 2016 to I_FER_ = 2.54 in 2015 (Figure 1), and the lowest mean degree of maize cob infection was noticed in Radzików (I_FER_ = 1.72). It was significantly higher in Smolice (I_FER_ = 1.96) and Kobierzyce (I_FER_ = 2.07) (Figure 1).

Despite the significant differences in the mean disease severity among the locations during the four-year study, the highest number of the tested breeding lines exhibited a low (I_FER_ = 2) and moderate (I_FER_ = 3) infection level. In 2015–2018, the value of the disease severity index (I_FER_) was greater than 1 and lower than 3 (according to the 6-degree scale) in the case of 76.68%, 84.50%, and 79.49% of the tested breeding lines cultivated in Kobierzyce, Smolice, and Radzików, respectively (Table 1).

### 2.2. Mycotoxin Occurrence

**Figure 2:**
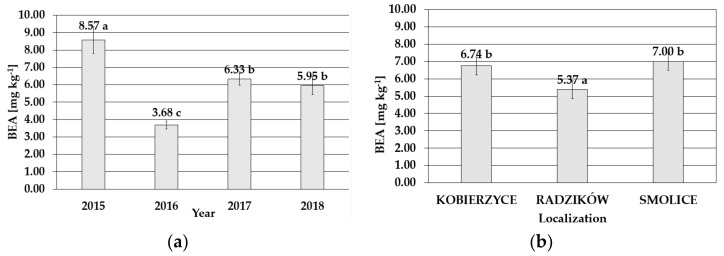
Mean BEA content with standard deviation bars in maize genotypes inoculated with *Fusarium temperatum* in the field test (**a**) during the four cropping seasons (2015–2018) and (**b**) during the four cropping seasons (2015–2018) in Smolice, Radzików, and Kobierzyce. The average values obtained within an individual cropping season for all genotypes of maize marked with the same letter do not differ significantly at *p* ≤ 0.05. The average values obtained for an individual localization over the all years of study marked with the same letter do not differ significantly at *p* ≤ 0.05.

Kernels from cobs infected with *F. temperatum* were contaminated with beauvericin (BEA), and the accumulation of this secondary metabolite predicted on raw data ranged from 0.00 to 103.59 mg kg^−1^, with average content of 6.13 mg kg^−1^ for four years. The lowest mean toxin accumulation was detected in 2016 (3.68 mg kg^−1^), while the BEA concentration in 2015, when the FER occurrence resulted in substantial maize damage, was more than two times higher, i.e., 8.57 mg kg^−1^ (Figure 2). The level of BEA correlated also with FER severity in particular localities. The lowest mean level of this metabolite was determined in maize cultivated in Radzików (5.37 mg kg^−1^). It was significantly higher in Kobierzyce (6.74 mg kg^−1^) and Smolice (7.00 mg kg^−1^) (Figure 2).

### 2.3. Maize Type

**Figure 3:**
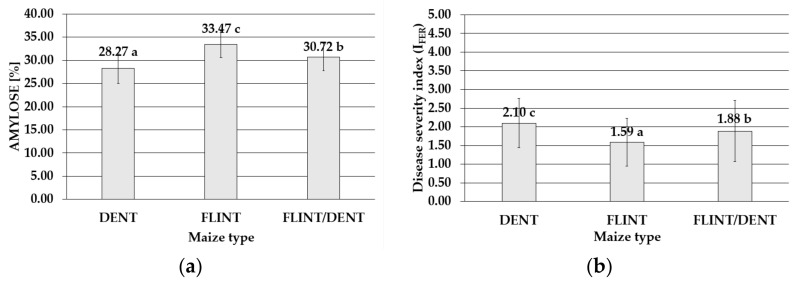
Mean with standard deviation bars (**a**) amylose content in the dent, flint, and flint/dent maize types in the field test during the four cropping seasons (2015–2018); (**b**) value of the disease severity index of the dent, flint, and flint/dent maize types inoculated with *Fusarium temperatum* in the field test during the four cropping seasons (2015–2018). The average values obtained within amylose content in the dent, flint, and flint/dent maize types marked with the same letter do not differ significantly at *p* ≤ 0.05. The average values obtained for the disease severity index of the dent, flint, and flint/dent maize types marked with the same letter do not differ significantly at *p* ≤ 0.05.

The ANOVA analysis showed a highly significant influence of the maize type on the FER severity index (Supplementary Table S1) and the content of BEA (Supplementary Table S2), ergosterol (ERG) (Supplementary Table S3), and amylose (Supplementary Table S4). In the samples of the maize genotypes tested during the four-year study, the amylose levels ranged from 20.28% to 41.13%, with a mean value of 30.78%. On average, the significantly highest concentrations of this starch fraction were found in the flint maize (33.47%). Lower content was detected in the flint/dent genotype (30.72%), and the lowest level was exhibited by the dent maize (28.27%) (Figure 3).

The most destructive *F. temperatum* impact was noticed in the dent genotypes, with the mean value of the disease severity index I_FER_ = 2.10 during the four study years (2015–2018). In the same period, the disease severity index of the flint maize population was significantly lower (I_FER_ = 1.59), while the flint/dent subpopulation exhibited an intermediate disease severity index (I_FER_ = 1.88) (Figure 3).

**Figure 4:**
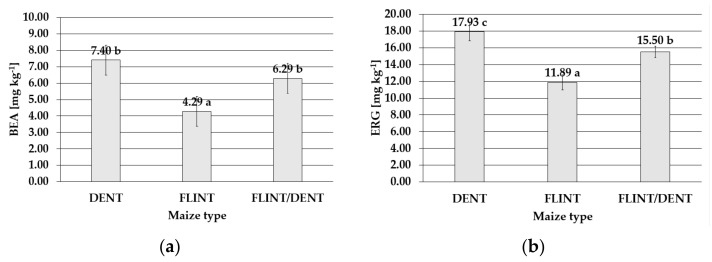
Mean content with standard deviation bars of (**a**) beauvericin in the dent, flint, and flint/dent maize types inoculated with *Fusarium temperatum* in the field test during the four cropping seasons (2015–2018); (**b**) ergosterol in the dent, flint, and flint/dent maize types inoculated with *Fusarium temperatum* in the field test during the four cropping seasons (2015–2018). The values obtained within mean content of (**a**) beauvericin and (**b**) ergosterol in the dent, flint, and flint/dent maize types marked with the same letter do not differ significantly at *p* ≤ 0.05.

The level of BEA was also maize-type dependent and reflected the I_FER_ severity. The lowest mean level of this metabolite was found in the flint maize (4.29 mg kg^−1^), and significantly higher content was detected in the dent maize type (7.40 mg kg^−1^) (Figure 4). Of the three examined maize subpopulations, the significantly highest ergosterol concentration was found in the dent kernels (17.93 mg kg^−1^). Lower content was detected in the flint/dent maize (15.50 mg kg^−1^), and the lowest level was determined in the flint type (11.89 mg kg^−1^) (Figure 4).

The conducted research indicated that the risk of kernel infection and contamination with fungal metabolites (beauvericin and ergosterol) was associated with the maize type kernels, which also differed significantly in the starch composition in the endosperm.

The estimated Pearson correlation coefficients showed a significant (*p* < 0.0001) relationship between the analyzed variables (Table 2). The most robust relationships were found between the disease severity index I_FER_ and the mycotoxin and ergosterol levels. Depending on the cropping seasons, the correlation coefficients (R) ranged from R = 0.396 in 2015 to R = 0.735 in 2018 between I_FER_ and ERG and from R = 0.364 in 2015 to R = 0.785 in 2017 between I_FER_ and BEA. The correlations between BEA and ERG also depended on the seasons and ranged from R = 0.486 to R = 0.911 in 2015 and 2017, respectively.

In the case of amylose, a significant correlation was found between amylose and I_FER_ only; however, these associations were weak (R = −0.303 to R= −0.180) depending on the year. The R values between the other factors (amylose vs. ERG and amylose vs. BEA) were not significant in all years of the studies, and the strength of the associations was weak.

It is worth emphasizing that the interaction effects between the maize type vs. the year and the maize type vs. the locations were not significant according to the ANOVA analysis, indicating stable classification of the plant maize types in regard to their infection level and the content of beauvericin (BEA), ergosterol (ERG), and amylose in the cropping seasons and locations (Table 3).

## 3. Discussions

Fusarium ear rot is one of the most devastating and serious diseases of maize worldwide [33,34]. Outbreak of this disease can result in kernels damage, their contamination by mycotoxins, and yield loss [35]. In the United States and Ontario, Canada, occurrence of FER caused yield losses of 349.2 million bushels in the period 2012–2015 and contributed to mycotoxin contamination estimated at the level of 4529.1 million bushels for maize kernels damaged with toxigenic species [36]. Among a large number of species responsible for FER, the significance of *Fusarium verticillioides* has been studied most comprehensively [37,38,39]. In the current studies, we focused on maize infection with *Fusarium temperatum.*

Although *F. temperatum* is reported as a new emerging fungal species, it has been recognized as a dangerous maize pathogen infecting the host in different plant growth development stages and reported as the cause of seed rot, seedling blight [40], root rot [41], and stalk rot [42]. Our results stress its role as a causative factor of ear rot of maize. In the infection assays performed (using the nail punch inoculation method) in 2015–2018, trace levels to 45% of kernels per ear were infected. In comparison with other ear-rot-associated *Fusaria*, the aggressiveness of *F. temperatum* was reported to be the closest to that of *F. subglutinans* and, to a lesser extent, to other less pathogenic taxons of the *Fusarium fujikuroi* species complex (*F. verticllioides, F. proliferatum*), which was proven in a toothpick inoculation assay, a seedling test [12], and a silk channel assay [5]. The most damaging effect on maize is exerted by *F. graminearum* and *F. culmorum*, i.e., species that are formally responsible for red ear rot. Depending on the research, *F. graminearum* [43,44] or *F. culmorum* [45] are reported as the most pathogenic species. The results of long-term, multi-location field studies using 404 genotypes proved a relationship between the level of *Fusarium* ear rot and the kernel maize type. The most destructive *F. temperatum* effects were noticed in the maize with the dent-type endosperm, and the estimated FER index significantly correlated with ergosterol (R = 0.396 to R = 0.735), BEA (R = 0.364 to R = 0.785), and amylose (R = −0.303 to R= −0.180) content.

The maize infection with *F. temperatum* resulted in BEA contamination of the kernels. The levels of this metabolite, based on raw data, ranged from 0.00 to 103.59 mg kg^−1^, with average content of 6.13 mg kg^−1^ and significantly different mean concentrations between the dent (7.40 mg kg^−1^) and flint (4.29 mg kg^−1^) maize kernels in the four-year study. BEA is reported as one of the most predominant mycotoxins in grains, and its concentration in visibly infected maize kernels can reach substantial levels. In Italian maize samples infected with *F. proliferatum*, the maximum BEA level was as high as 520 mg kg^−1^ [46], while the BEA concentration in maize collected from Greater Poland and contaminated with *F. subglutinans* and *F. verticillioides* reached 1731.55 mg kg^−1^ [47]. The mean BEA concentration detected in the maize kernels in the present study was one to two orders of magnitude lower than the extremes reported previously [46,47].

The importance of maize endosperm in relation to FER resistance and mycotoxin contamination was originally raised by Snijders [48], who found no significant effect of this part of kernels on maize resistance to *Fusarium*. Similarly, Shelby et al. [49] did not find any significant correlation between the composition of maize kernels (starch, lipid, fiber, and protein content) and fumonisin levels. In turn, Bluhm and Woloshuk [50] proved that kernels lacking starch due to physiological immaturity do not accumulate FB1, and significantly less FB1 is produced in a high-amylose maize kernel mutant. The comparative studies of flint and dent genotypes revealed little about the endosperm as an important factor for FER resistance. Hennigen et al. [51] did not prove any significant differences in the cob infection degree between the flint and dent maize genotypes. Higher disease severity in maize with the flint endosperm type was observed by Löffler et al. [52]. Our findings stress the contribution of kernel endosperm in host response to FER caused by *F. temperatum* and correspond to a previous paper by Wit et al. [43] reporting higher susceptibility of dent maize (*Zea mays* var. *indentata*) to common *Fusarium* species occurring on maize ears (*F. avenaceum*, *F. culmorum*, *F. equiseti*, *F. graminearum*, *F. proliferatum*, *F. subglutinans*, and *F. verticillioides*).

In this study, both type kernels differed in the starch component composition; a significantly higher amylose level was noticed in the flint (33.47%) versus dent type, which contained only (28.27%). Amylopectin and amylose are the predominant components of maize starch granules. Amylopectin has a highly branched structure composed of (1, 4)-linked glucose linear chains and α(1–6)-linked branch points [53], while amylose is a linear polymer of glucose molecules and is linked by α-l,4 glycosidic linkages, which makes this compound of starch less susceptible to enzymatic digestion [54]. For this reason, amylose is recognized as a factor that slows down the digestion rate and the digestibility of starch granules [55], which may have contributed to the less severe flint kernel damage by *F. temperatum* than in the dent maize observed in our study. Several other factors, e.g., pericarp layer thickness, kernel hardness, and phenolic compound contents, are associated with maize kernel response (resistance) to *Fusarium*; however, the mutual interactions among these multi-gene traits are not satisfactorily elucidated.

## 4. Materials and Methods

### 4.1. Experimental Design

Surveys were conducted to assess if there were significant differences in infection degree and beauvericin contamination of flint, dent, and intermediate flint/dent maize types inoculated with *Fusarium temperatum* in relation to endosperm starch composition. Infection assay were carried out outdoors in three locations during four cropping seasons. Each year, after silks emergence, plants were inoculated with the selected 5 highly aggressive isolates of *F. temperatum.* After harvest infection degree, kernel contamination by beauvericin and amylose content were determined.

### 4.2. Plant Materials

Three maize populations differing in morphological features and representing *Zea mays* var. *indentata*, *Zea mays* var. *indurata*, and plants with intermediate kernel characteristics were included in the studies. The breeding lines of both botanical varieties used in the research were developed and provided by Smolice Plant Breeding LTD and Malopolska Plant Breeding LTD.

The pool of the tested germplasm consisted of heterotic material and various kinds of elite inbred lines being the starting genetic material in the maize breeding process developed at Smolice Plant Breeding LTD (203) and Malopolska Plant Breeding LTD (201 genotypes).

The experiments were carried out from 2015 to 2018 in three locations in Poland: Plant Breeding and Acclimatization Institute (IHAR)—National Research Institute in Radzików (52°13′9.444″ N 20°37′52.949″ E), Smolice Plant Breeding LTD (51°42′20.466″ N 17°9′57.241″ E), and Malopolska Plant Breeding LTD in Kobierzyce (50°58′19.411″ N 16°55′47.323″ E). The trials were set up in a randomized complete block with two replications. Each replicate consisted of a 1-row plot (8 m long) with at least 11 plants.

### 4.3. Plant Infection Assay

The plants were inoculated at 10 days [56] after silk emergence outside the husks, just before the R2 blister maize growth stage, when the kernels become white with clear liquid inside [57]. The inoculum was introduced to the cob through the husk midway between the base and ear tip, according to the nail punch method [58].

The ratings of the disease severity were performed at the end of the growing season. Inoculated ears were harvested at maturity, transported to the laboratory, dried, and then visually evaluated for FER according to a 6-point disease severity index (I_FER_) related to the percentage of kernels per ear with FER symptoms (Symptomatic Kernels SK) (0–no symptoms, 1–very low infection: up to 3% of SK, 2–low infection: 3 to 10% of SK, 3–moderate infection: 10 to 30% of SK, 4–severe infection: 30 to 50% of SK, 5–very severe infection: over 50% of SK) as described by Wit et al. [59].

### 4.4. Inoculum Preparation

The inoculum was prepared on the basis of 5 highly aggressive isolates of *F. temperatum* deposited in the culture collection of the Department of Plant Protection, Section of Plant Pathology, Institute of Horticultural Sciences, Warsaw University of Life Sciences (WULS-SGGW). Originally, the *F. temperatum* strains were obtained by direct isolation from rotten cob tissues exhibiting typical etiological infection symptoms of FER or by transfer of infected kernels onto synthetic nutrient-poor agar (SNA) medium (Difco^TM^, Sparks, MD, USA). After development of colonies, the mycelia of the fungi were passaged on agar slants using the hyphal tip technique. Mycological analysis was performed according to the Leslie and Summerell [60] system. The taxonomy of the strains was confirmed by DNA barcode analysis with the use of *EF-1α* and *β-tubulin* genes as markers as described by Jabłońska et al. [2]. All mycological and molecular studies were carried out using single spore isolates obtained previously [61]. The aggressiveness of the *F. temperatum* strains was confirmed in a pathogenicity test conducted with the toothpick inoculation method according to Scauflaire et al. [12]. Finally, for inoculation tests, the isolates were grown on potato dextrose agar (PDA) medium (Difco^TM^, Sparks, MD, USA) in Petri dishes and incubated for 10 days at 22 °C as in Kwaśna et al. [62]. The aerial mycelium was scraped, suspended in distilled water, and filtered through cheesecloth. The concentration of the spore suspension was adjusted to 10^6^ cfu per 1 mL.

### 4.5. Mycotoxin Analysis

Organic solvents: acetonitrile and methanol (HPLC grade) were purchased from POCH (Gliwice, Poland). Water for the HPLC analysis was purified in a Milli-Q Academic system (Millipore S.A.S., Molsheim, France).

Beauvericin (BEA) was extracted by shaking 10 g of an inoculated maize ground sample in 15 mL of methanol on a compact shaker KS-15 (Edmund Bühler, Germany) for 30 min at 350 rpm. The samples were then centrifuged for 5 min at 3500 rpm, and 2 mL of clear methanol extract was transferred to a 4 mL vial and evaporated in a stream of nitrogen. Dried extracts were resuspended in 1 mL of methanol and filtered through a syringe filter (Nylon 66, pore size 0.22 µm) before HPLC analysis. Beauvericin analyses were performed according to Monti et al. [63] and Logrieco et al. [64] with modifications. HPLC analyses were performed using a Perkin-Elmer Flexar HPLC (PerkinElmer, Inc., Waltham, MA, USA) system with a diode array detector and a Chromera chromatographic system (Perkin-Elmer, Sciex Instruments, Ontario, Canada).

Analytes were separated on a NovaPak C18 column, 150 × 4.6 mm, 4 µm (cat. No. WAT086344 Waters, Ireland) with a proper guard column. Separations were conducted at a constant flow of 1.3 mL min^−1^ and an aqueous acetonitrile solution as the eluent system. The starting acetonitrile:water ratio (68:32, *v/v*) was kept for 11 min and then linearly increased to 86% acetonitrile within 3 min, and after 10 min at 86% acetonitrile, the mobile phase was changed to the starting conditions within 4 min. Mycotoxins were detected at λ = 205 nm, identified by comparison of retention times, and confirmed by comparison of the UV spectra of the samples with pure standards. The content of mycotoxins was calculated based on the calibration curves of peak areas vs. the amount of injected standards. All analyses were run in duplicate, and the mean values are reported. Ergosterol (ERG) was analysed via high-performance liquid chromatography (HPLC), according to protocol presented by Waskiewicz et al. [65]. In brief, samples containing 100 mg of ground grains were suspended in 2 mL of methanol, treated with 0.5 mL of 2 M aqueous sodium hydroxide, and tightly sealed. Obtained suspension were irradiated (370 W) for 20 s and following approximately 5 min for an additional 20 s. After 15 min, the contents of the culture tubes were neutralized with 1 M aqueous hydrochloric acid and supplemented with 2 mL MeOH. Extraction of ergosterol were performed three times with 4 mL pentane. The combined pentane extracts were evaporated to dryness in a nitrogen stream. Precipitate was dissolved in 1 mL of MeOH, filtered through 13 mm syringe filters with a 0.45 µm pore diameter (Fluoropore Membrane Filters, Millipore, Corcaigh, Ireland), and 50 µL of sample were injected on HPLC column. Separation was performed on a reversed phase column Nova Pak C-18 (Waters, Milford, MA, USA), 150 × 3.9 mm, particle size 4 µm, and eluted with methanol:acetonitrile (90:10, *v/v*) at a flow rate of 0.6 mL min^−1^. Ergosterol was detected with a Waters 486 Tunable Absorbance Detector (Milford, MA, USA) set at 282 nm. The presence of ergosterol (ERG) was confirmed by a comparison of retention times and by co-injection of every tenth sample with an ergosterol standard.

### 4.6. Amylose Analysis

Amylose analyses were performed using a slightly modified procedure recommended by the kit producer (Megazyme, Wicklow, Ireland) as described by Waskiewicz et al. [65]. Flour samples (20–25 mg) obtained from healthy cob kernels were dispersed by heating (100 °C, 15 min) in 1 mL dimethyl sulphoxide (DMSO). Starch was precipitated twice with the use of 5 mL methanol (95%) to remove lipids. The probes were centrifuged, the supernatant was discarded, and starch was hydrolyzed in 2 mL DMSO in a thermoblock (100 °C, 15 min). Subsequently, hydrolyzed starch was supplemented with sodium acetate buffer (600 mM, pH 6.4) up to 25 mL of the solution volume. Concavalin was used to precipitate amylopectin in the starch solution. The obtained amylose (0.5 mL) was added to 1.5 mL of sodium acetate buffer (600 mM, pH 4.5) and incubated for 5 min at 100 °C. Glucose oxidase (200 U) and peroxidase (500 U) reagents (50 mL) were used to quantify the amylose content by hydrolysis to D-glucose. The content of amylose was estimated spectrophotometrically in a microtitration 96-well plate, and absorbance was measured at a wavelength of 510 nm. The mean concentration of each quantified compound was calculated on the basis of eight independent measurements.

### 4.7. Statistical Analysis

All the data were analyzed by StatisticaStatSoft Inc. The differences between the disease severity index and the content of beauvericin and ergosterol over the independent variables, i.e., maize type, localities, and years, were evaluated by analysis of variance at a significance level of 0.05. Differences between the means were identified in the post hoc comparison procedure using Tukey grouping of the least significant difference at α = 0.05. The relationships between the disease severity index and the content of ergosterol, beauvericin, and amylose were expressed by Pearson correlation coefficients.

## Figures and Tables

**Table 1:** Distribution of the disease severity index (I_FER_) of the maize breeding lines cultivated in Smolice, Kobierzyce, and Radzików in 2015–2018.

Disease Severity Index (I_FER_)	YEAR	KOBIERZYCE		RADZIKÓW		SMOLICE		SUM	
		Breeding Lines		Breeding Lines		Breeding Lines		Breeding Lines	
		No	%	No	%	No	%	No	%
0–1	2015	0	0.00	3	3.03	0	0.00	3	1.01
	2016	10	20.41	34	33.33	6	11.54	50	21.76
	2017	1	2.00	4	3.92	1	1.92	6	2.61
	2018	6	11.76	23	22.77	6	12.00	35	15.51
	2015–2018	17	8.54	64	15.76	13	6.37	94	10.22
1–2	2015	9	17.65	38	38.38	0	0.00	47	18.68
	2016	28	57.14	58	56.86	39	75.00	125	63.00
	2017	19	38.00	54	52.94	32	61.54	105	50.83
	2018	36	70.59	73	72.28	37	74.00	146	72.29
	2015–2018	92	45.85	223	55.12	108	52.64	423	51.20
2–3	2015	23	45.10	46	46.46	33	67.35	102	52.97
	2016	10	20.41	10	9.80	7	13.46	27	14.56
	2017	24	48.00	38	37.25	18	34.62	80	39.96
	2018	5	9.80	4	3.96	6	12.00	15	8.59
	2015–2018	62	30.83	98	24.37	64	31.86	224	29.02
3–4	2015	15	29.41	11	11.11	15	30.61	41	23.71
	2016	1	2.04	0	0.00	0	0,00	1	0.68
	2017	5	10.00	6	5.88	1	1.92	12	5.93
	2018	4	7.84	1	0.99	1	2.00	6	3.61
	2015–2018	25	12.32	18	4.50	17	8.63	60	8.48
4–5	2015	4	7.84	1	1.01	1	2.04	6	3.63
	2016	0	0.00	0	0.00	0	0.00	0	0.00
	2017	1	2.00	0	0.00	0	0.00	1	0.67
	2018	0	0.00	0	0.00	0	0.00	0	0.00
	2015–2018	5	2.46	1	0.25	1	0.51	7	1.07

**Table 2:** Pearson’s correlations (R) between the estimated variables: infection degree (I_FER_), ergosterol (ERG), beauvericin (BEA), and amylose content (AMYL) in the growing seasons 2015–2018.

Estimated Variables	YEAR	I_FER_	ERG	BEA	AMYL
**I_FER_**	2015	1.000	0.396 **	0.364 **	−0.195 **
	2016	1.000	0.441 **	0.430 **	−0.180 *
	2017	1.000	0.701 **	0.785 **	−0.201 **
	2018	1.000	0.735 **	0.503 **	−0.303 **
**ERG**	2015	0.396 **	1.000	0.486 **	−0.253 **
	2016	0.441 **	1.000	0.787 **	−0.028
	2017	0.701 **	1.000	0.911 **	−0.175 *
	2018	0.735 **	1.000	0.581 **	−0.322 **
**BEA**	2015	0.364 **	0.486 **	1.000	−0.091
	2016	0.430 **	0.787 **	1.000	−0.086
	2017	0.785 **	0.911 **	1.000	−0.160 *
	2018	0.503 **	0.581 **	1.000	−0.166 *
**AMYL**	2015	−0.195 **	−0.253 **	−0.091	1.000
	2016	−0.180 *	−0.028	−0.086	1.000
	2017	−0.201 **	−0.175 *	−0.160 *	1.000
	2018	−0.303 **	−0.322 **	−0.166 *	1.000

* and ** indicate significance at the 95% and 99.0% level, respectively.

**Table 3:** Infection degree I_FER_, ergosterol (ERG), and beauvericin (BEA) content in relation to amylose in maize inoculated with *Fusarium temperatum* in 2015–2018. The average values obtained for disease severity index I_FER_, ERG, BEA, and amylose respectively, marked with the same letter do not differ significantly at *p* ≤ 0.05.

Year	Maize Type	No of Tested Breeding Lines	Disease Severity Index I_FER_	ERG (mg kg^−1^)	BEA (mg kg^−1^)	Amylose (%)
2015	D	38	2.758 ± 0.576 ^b^	14,083 ± 12.68 ^a^	12,219 ± 8.83 ^b^	25.319 ± 2.38 ^a^
	F	40	2.200 ± 0585 ^a^	6822 ± 11.71 ^ab^	4563 ± 5.41 ^a^	30.742 ± 2.37 ^c^
	FD	121	2.578 ± 0.790 ^b^	10,611 ± 15.32 ^b^	8780 ± 12.20 ^b^	28.065 ± 2.46 ^b^
2016	D	40	1.692 ± 0.513 ^c^	17,630 ± 11.80 ^a^	3716 ± 2.40 ^a^	29.422 ± 2.82 ^a^
	F	40	1.194 ± 0.439 ^a^	16,487 ± 15.25 ^a^	3419 ± 3.83 ^a^	33.758 ± 2.34 ^c^
	FD	123	1.432 ± 0.644 ^b^	16,616 ± 13.60 ^a^	3745 ± 3.30 ^a^	31.149 ± 2.13 ^b^
2017	D	40	2.211 ± 0.426 ^b^	20,047 ± 11.01 ^b^	6987 ± 3.89 ^b^	29.779 ± 3.24 ^a^
	F	40	1.679 ± 0.554 ^a^	12,042 ± 6.51 ^a^	4507 ± 2.86 ^a^	35.120 ± 2.14 ^c^
	FD	124	2.083 ± 0.680 ^b^	19,551 ± 15.32 ^b^	6702 ± 5.79 ^b^	32.552 ± 2.73 ^b^
2018	D	40	1.767 ± 0.519 ^b^	19,665 ± 17.39 ^ab^	7024 ± 6.63 ^a^	28.413 ± 2.42 ^a^
	F	40	1.278 ± 0.380 ^a^	12,189 ± 8.86 ^a^	4733 ± 3.06 ^a^	34.255 ± 2.65 ^c^
	FD	122	1.443 ± 0.531 ^a^	15,101 ± 16.00 ^b^	5949 ± 7.49 ^a^	31.082 ± 2.63 ^b^
2015–2018	D	158	2.099 ± 0.659 ^c^	17,928 ± 13.54 ^b^	7401 ± 6.58 ^b^	28.270 ± 3.23 ^a^
	F	160	1.588 ± 0.634 ^a^	11,885 ± 11.50 ^a^	4286 ± 3.95 ^a^	33.469 ± 2.88 ^c^
	FD	490	1.883 ± 0.820 ^b^	15,501 ± 15.38 ^ab^	6289 ± 8.06 ^b^	30.721 ± 2.98 ^b^

Abbreviations: D, dent maize type; F, flint maize type; FD, flint/dent maize type.

## Data Availability

The data presented in this study are available in this article, supplementary materials and from the corresponding author.

## Supplementary Materials

The following supporting information can be downloaded at: https://www.mdpi.com/article/10.3390/toxins14030200/s1, Table S1: ANOVA analysis of the influence of the maize type, cropping season, and field localization on disease severity index I_FER_ after plant inoculation with *F. temperatum*; Table S2: ANOVA analysis of the influence of the maize type, cropping season, and field localization on BEA kernel contamination after plant inoculation with *F. temperatum*; Table S3: ANOVA analysis of the influence of the maize type, cropping season, and field localization on the ergosterol (ERG) content after plant inoculation with *F. temperatum*; Table S4: ANOVA analysis of the influence of the maize type, cropping season, and field localization influence on the amylose content in maize kernels.
