# Supplementary material for: Influence of Endosperm Starch Composition on Maize Response to Fusarium temperatum Scaufl. & Munaut
Source: Toxins (Basel). 2022 Mar 8;14(3):200. doi: 10.3390/toxins14030200 (PMC8951129; doi:10.3390/toxins14030200)
Supplement: Supplementary file 1 [file toxins-14-00200-s001.zip › toxins-1606313-supplementary.pdf]

# Supplementary Materials: Influence of Endosperm Starch Composition on Maize Response to Fusarium temperatum Scaufl. & Munaut

**Marcin Wit, Piotr Ochodzki, Roman Warzecha, Emilia Jabłońska, Ewa Mirzwa-Mróz, Elżbieta Mielniczuk, and Wojciech Wakuliński**

**Table S1.** ANOVA analysis of the influence of the maize type, cropping season, and field localization on disease severity index $I_{FER}$ after plant inoculation with *F. temperatum.*

| **Source of variation** | **ANOVA traits** | | | | |
|---|---|---|---|---|---|
| | *SS* | *df* | *MS* | *F* | *p* |
| Intercept | 140875.1 | 1 | 140875.1 | 785.1575 | 0.000000 |
| Maize type (A) | 2865.9 | 2 | 1433.0 | 7.9865 | 0.000369 |
| Year (B) | 3847.8 | 3 | 1282.6 | 7.1485 | 0.000097 |
| Localities (C) | 8551.7 | 2 | 4275.8 | 23.8310 | 0.000000 |
| A X B | 1350.7 | 6 | 225.1 | 1.2547 | 0.276174 |
| A X C | 754.3 | 4 | 188.6 | 1.0510 | 0.379854 |
| B X C | 4428.8 | 6 | 738.1 | 4.1139 | 0.000448 |
| A X B X C | 1249.6 | 12 | 104.1 | 0.5804 | 0.859055 |
| Error | 138514.4 | 772 | 179.4 | | |

Abbreviations: *SS*, sum of squares; *df*, degrees of freedom; *MS*, Mean square; *F*, F ratio; *p*, probability.

**Table S2.** ANOVA analysis of the influence of the maize type, cropping season, and field localization on BEA kernel contamination after plant inoculation with *F. temperatum.*

| **Source of variation** | **ANOVA traits** | | | | |
|---|---|---|---|---|---|
| | *SS* | *df* | *MS* | *F* | *p* |
| Intercept | 20874.16 | 1 | 20874.16 | 454.6599 | 0.000000 |
| Maize type (A) | 780.60 | 2 | 390.30 | 8.5011 | 0.000223 |
| Year (B) | 1795.12 | 3 | 598.37 | 13.0332 | 0.000000 |
| Localities (C) | 241.12 | 2 | 120.56 | 2.6259 | 0.073039 |
| A X B | 652.21 | 6 | 108.70 | 2.3676 | 0.028426 |
| A X C | 365.52 | 4 | 91.38 | 1.9903 | 0.094177 |
| B X C | 936.68 | 6 | 156.11 | 3.4003 | 0.002569 |
| A X B X C | 211.52 | 12 | 17.63 | 0.3839 | 0.969385 |
| Error | 34479.60 | 751 | 45.91 | | |

Abbreviations: *SS*, sum of squares; *df*, degrees of freedom; *MS*, Mean square; *F*, F ratio; *p*, probability.

**Table S3.** ANOVA analysis of the influence of the maize type, cropping season, and field localization on the ergosterol (ERG) content after plant inoculation with *F. temperatum.*

| Source of variation | ANOVA traits | | | | |
|---|---|---|---|---|---|
| | ***SS*** | ***df*** | ***MS*** | ***F*** | ***p*** |
| Intercept | 140875.1 | 1 | 140875.1 | 785.1575 | 0.000000 |
| Maize type (A) | 2865.9 | 2 | 1433.0 | 7.9865 | 0.000369 |
| Year (B) | 3847.8 | 3 | 1282.6 | 7.1485 | 0.000097 |
| Localities (C) | 8551.7 | 2 | 4275.8 | 23.8310 | 0.000000 |
| A X B | 1350.7 | 6 | 225.1 | 1.2547 | 0.276174 |
| A X C | 754.3 | 4 | 188.6 | 1.0510 | 0.379854 |
| B X C | 4428.8 | 6 | 738.1 | 4.1139 | 0.000448 |
| A X B X C | 1249.6 | 12 | 104.1 | 0.5804 | 0.859055 |
| Error | 138514.4 | 772 | 179.4 | | |

Abbreviations: *SS*, sum of squares; *df,* degrees of freedom; *MS*, Mean square; *F*, F ratio; *p*, probability.

**Table S4.** ANOVA analysis of the influence of the maize type, cropping season, and field localization influence on the amylose content in maize kernels.

| Source of variation | ANOVA traits | | | | |
|---|---|---|---|---|---|
| | ***SS*** | ***df*** | ***MS*** | ***F*** | ***p*** |
| Intercept | 519879.4 | 1 | 519879.4 | 94644.65 | 0.000000 |
| Maize type (A) | 2052.4 | 2 | 1026.2 | 186.82 | 0.000000 |
| Year (B) | 1294.4 | 3 | 431.5 | 78.55 | 0.000000 |
| Localities (C) | 220.6 | 2 | 110.3 | 20.08 | 0.000000 |
| A X B | 32.6 | 6 | 5.4 | 0.99 | 0.431839 |
| A X C | 51.6 | 4 | 12.9 | 2.35 | 0.052858 |
| B X C | 145.2 | 6 | 24.2 | 4.40 | 0.000217 |
| A X B X C | 246.9 | 12 | 20.6 | 3.75 | 0.000015 |
| Error | 4251.6 | 774 | 5.5 | | |

Abbreviations: *SS*, sum of squares; *df,* degrees of freedom; *MS*, Mean square; *F*, F ratio; *p*, probability.
